# Intervening young children with road safety education yields reduction in road crashes 

**Published:** 2019-08

**Authors:** Kulanthayan KC Mani, Hussain Hamid, Ahmad Hariza Hashim, Law Teik Hua

**Affiliations:** ^*a*^Safe Kids Malaysia, Universiti Putra Malaysia, Malaysia.; ^*b*^Road Safety Research Centre, Universiti Putra Malaysia, Malaysia.

## Abstract

**Background:**

Road Safety Education (RSE) intervention is a joint effort between Ministry of Transport Malaysia and Ministry of Education Malaysia. RSE was implemented in stages from 2007 to nationwide rollout in all standards and in all primary schools in the country by 2010. The RSE knowledge is embedded in the existing curriculum through the National Malay Language subject. RSE was taught once a week (40 minutes exposure) in the classroom by the subject teachers whom have undergone special 3 days training on RSE module. The RSE modules are developed appropriately tailored to the age-range of the students. Objective: To evaluate the effectiveness of RSE intervention before and after implementation.

**Methods:**

A prospective intervention-control study following children who are exposed and not exposed to RSE program for 2 years and observing whether they are involved in road traffic injuries (RTI) over the period of time. Children with intervention in this study will be taken from schools where new RSE program is to be implemented. There will be matched controls from neighboring districts with schools where program not implemented. This gives a ratio of 1:1 between intervention and control. For this study, Quasi Experimental-interrupted time series design with comparison group was applied since random allocation was not possible. Six intervention districts and six comparison districts was selected as per Education District List under the Ministry of Education Malaysia. Total sample size of 67,232 children for both years involved in study (33,616 per arm). Minimum sample of 33,616 children from 6 intervention districts and 33,616 children from control districts of Standard 2 (age 8) and 4 (age 10) were required.

**Results:**

Health Outcome based study was conducted for two years (2008-2009) for different targets groups. Total students sampled of 20,396 among year 2 (age 8) and 19,721 year 4 (age 10). from injury surveillance study showed a significant reduction in number of crashes in intervention districts with RSE program compared to control districts without RSE program for both year 2 (age 8) and 4 (age 10) students after following up for two years. Next, results from police crash data showed a reduction in number of road crashes for pedestrian age group 7-12 years in intervention districts as compared to control districts comparing year 2007 and 2009.

**Conclusions:**

The study has shown from the injury surveillance study, a declining trend in number of children road crashes recorded for year 2 (age 8) and 4 (age 10) students whom receive RSE intervention as compared to those who did not receive the intervention. The study findings has managed to convince both main stakeholders MOT and MOE to rollout the intervention nationwide to all primary schools from 2010 onwards and secondary schools later.

**Keywords:**

Road Safety Education Initiative, Road Crash, Children

